# Supplementary material for: Suppression of pathogens in properly refrigerated raw milk
Source: PLoS One. 2023 Dec 12;18(12):e0289249. doi: 10.1371/journal.pone.0289249 (PMC10715650; doi:10.1371/journal.pone.0289249)
Supplement: S1 Appendix — (PDF) [file pone.0289249.s001.pdf]

FEBRUARY 09, 2022

FULL REPORT

Determination of Growth Rate of *Salmonella enterica* spp., *E. coli* O157:H7, *Campylobacter* spp., and *Listeria monocytogenes* in Raw Milk

PREPARED FOR:

Raw Milk Institute

TABLE OF CONTENTS

1.0 FINAL DATES 3
2.0 PARTIES 3
3.0 OBJECTIVE 3
4.0 MATERIALS AND METHODS 3
    4.1 Receipt of Samples 3
    4.2 Inoculation of Samples 4
    4.3 Analysis of Inoculated Samples 6
    4.4 Analysis of Uninoculated Samples 6
5.0 RESULTS 7
6.0 CONCLUSION 13
7.0 FINAL REPORT APPROVAL 14

1.0 FINAL DATES

Project Initiation Date: May 18, 2021
Date Testing Began: August 04, 2021
Date Testing Completed: January 01, 2022
Project Completion Date: Date of Final Report Approval
Study Authorization: Signed Proposal

2.0 PARTIES

Project Sponsor: Raw Milk Institute (RAWMI)

3.0 OBJECTIVES

Raw Milk Institute (RAWMI) reached out to conduct a study to determine the growth of four foodborne pathogens, namely *Salmonella enterica* spp., *E. coli* O157:H7, *Campylobacter* spp., and *Listeria monocytogenes* in raw milk stored at 4.4 ± 1°C over 14 days period. More specifically, RAWMI desired to determine the growth rate of the above pathogens in raw milk. **The objective of this study was to inoculate raw milk provided by RAWMI and determine the growth of these pathogens when stored at 4.4 ± 1°C over 14 days.** As per RAWMI, the shelf life of the raw milk is seven days but they wanted to evaluate the potential growth of these pathogens over 14 days. The obtained data will be used in a predictive microbiology with the study outlined below. This study was conducted based on the study design provided by RAWMII.

4.0 MATERIALS AND METHODS

4.1 Receipt of Samples

To address this objective, RAWMI conducted a study on three different lots of raw milk. Upon receipt of the first set of three lots of milk, the outlined study was conducted on *Salmonella enterica* spp., *E. coli* O157:H7, and *Listeria monocytogenes* only. Survival/growth on *Campylobacter* strains could not be started at the same time because some of the Campylobacter strains did not grow adequately on the day of the receipt of the raw milk samples. It was decided that the study with Campylobacter strains would be carried out on separate lots of raw milk at a later date. Hence, the study was completed on two separate shipments each containing three different lots of milk. For each replicate of the study, RAWMI sent half a gallon of raw milk. Each gallon of milk was used as two technical replicates. The breakdown of testing is shown in Tables 1 and 2.

[THE REMAINDER FO THIS PAGE IS INTENTIONALLY LEFT BLANK]

Table 1. Lots of Milk To be Tested as Three Biological Replicates with Two Technical Replicates.

Shipment/Pathogens	Milk Lots	Technical Rep 1	Technical Rep 2
First shipment:<br>*Salmonella* spp.<br>*L. monocytogenes*<br>*E. coli* O157:H7<br>Indicator Organisms	A	x	x
	B	x	x
	C	x	x
Second shipment:<br>*Campylobacter* spp.<br>Indicator Organisms	A	x	x
	B	x	x
	C	x	x

Table 2. Test Conditions for Each Biological Replicates.

Lot A	*Salmonella* spp.,	*L. Monocytogenes*	*E. coli* O157:H7	*Campylobacter* spp.,
Technical Rep 1	-Inoculum level I<br>-Inoculum level II	-Inoculum level I<br>-Inoculum level II	-Inoculum level I<br>-Inoculum level II	-Inoculum level I<br>-Inoculum level II
Technical Rep 2	-Inoculum level I<br>-Inoculum level II	-Inoculum level I<br>-Inoculum level II	-Inoculum level I<br>-Inoculum level II	-Inoculum level I<br>-Inoculum level II

Note: The same sample set up was done on samples from Lots B and C. The 50 ml portions were inoculated with each pathogen type at Inoculum level I (target <10 CFU/ml) and Inoculum level II (target ~1,000 CFU/ml). A separate 50 ml sample portion was used for pH measurement and other indicator microbiological analysis at the start and end of the study. Additional milk samples were inoculated with *Campylobacter* species to determine presence/absence over 14 days of storage.

4.2 Inoculation of Samples

Inoculation of samples was conducted on the day of the receipt of milk. For each pathogen, separate cocktails of strains were prepared for the inoculation of the raw milk samples. Prior to inoculation, the raw milk samples were weighed out in 50 ml portions to be inoculated at two inoculum levels for each pathogen type: *Salmonella enterica* spp., *E. coli* O157:H7, *Campylobacter* spp., and *Listeria monocytogenes.* Additional sample portions in 50 ml aliquots were inoculated to be tested for the presence/absence of *Campylobacter* species. For the preparation of inoculum, three strains of each organism (listed below) were used to prepare the cocktail for each pathogen.

For *Salmonella enterica* Spp.:

- *Salmonella enterica* serotype Typhimurium ATCC 14028, Food Isolate
- *Salmonella enterica* serotype Seftenberg 775W ATCC 43845, Food Isolate
- *Salmonella enterica* serotype Enteritidis ATCC 49218

For *Listeria monocytogenes:*

- *Listeria monocytogenes* ATCC 19115, Serotype 4b, Human Isolate
- *Listeria monocytogenes* ATCC 19114, Serotype 4a, Animal Isolate
- *Listeria monocytogenes* ATCC 7644, Serotype 1/2c, Human Isolate

For *E. coli* O157:H7:

- *Escherichia coli* O157:H7 ATCC 700599, Salami Product Isolate
- *Escherichia coli* O157:H7 ATCC 35150, Human feces Isolate
- *Escherichia coli* O157:H7 ATCC 43895, Raw Hamburger Meat Isolate

For *Campylobacter* spp:

- *Campylobacter jejuni* ATCC 33291
- *Campylobacter jejuni* ATCC 33560
- *Campylobacter coli* ATCC 33559

For the preparation of the bacterial inoculum, fresh cultures of each strain were prepared by streaking frozen stock cultures (-80°C) onto Tryptic Soy Agar (TSA; Becton, Dickinson and Company, Franklin Lakes, NJ) and incubating at 35 ± 2°C for 21 ± 3 h. An isolated colony from each TSA plate was inoculated into a fresh 10 ml tube of Tryptic Soy Broth (TSB; Becton, Dickinson and Company) and incubated at 35 ± 2°C for 21 ± 3 h. For *Campylobacter* spp, an isolated colony was streaked onto Horse Blood Agar and incubated at 42°C for 48 h under microaerophilic conditions. Post incubation, an isolated colony was transferred to 1X Bolton broth containing 5% laked horse blood and incubated in vented vials in microaerophilic conditions for 48 h at 42°C. Aliquots of the incubated TSB or Campy culture of each strain were harvested by centrifugation at maximum speed for 10 min in an Avanti J-15 Centrifuge (Beckman Coulter, Inc., Pasadena, CA). The resultant supernatant was removed, and the pelleted cells were re-suspended in Butterfield's Phosphate Buffer (BPB; Made In-House From Various Ingredients). The re-suspended cells in BPB were centrifuged once more, followed by removal of supernatants, and re-suspension of the pelleted cells in BPB to wash off any leftover nutrient media. The resulting bacterial culture was mixed together and vortexed to distribute each strain evenly throughout, and obtain a three-strain cocktail. This three-strain cocktail was adjusted at ~8.0 $\log_{10}$ CFU/ml using a Spectrophotometer (Shimadzu, Columbia, MD). The cocktail was placed into 4.4 ± 1°C storage for a minimum of 10 h in order to cold stress the cells. The concentration of cells in this cold-stressed cocktail was also be verified via enumeration using the procedures described below. The cold-stressed 8.0 $\log_{10}$ CFU/ml cocktail was serially diluted to inoculate each 50 ml sample portion.

To determine the presence/absence of *Campylobacter* spp., two additional 50 ml portions for each lot of milk were inoculated at Inoculum level I and Inoculum level II. Two 50 ml portions were used at each time point (day 0, 3, 6, 9, 12, and 14) to test for the presence/absence of *Campylobacter* spp. from each lot of milk at each inoculum level.

4.3 *Analysis of Inoculated Samples*

Enumeration of pathogens were conducted on days 0, 3, 6, 9, 12, and 14. The presence/absence of *Campylobacter* spp. was also conducted at these time points on separate portions of samples inoculated with *Campylobacter* spp. At each test time point, the inoculated raw milk portions were shaken and one ml was retrieved for enumeration of the specific pathogen. One ml sample was serially diluted in BPB and spread-plated using selective agar plates. Xylose Lysine Deoxycholate Agar (XLD), Modified Oxford Agar (MOX), and Sorbitol MacConkey Agar with Cefixime and Tellurite (CT-SMAC) were used for the enumeration of *S. enterica, L. monocytogenes,* and *E. coli* O157:H7, respectively. XLD and CT-SMAC agar plates were incubated at 35°C for 24 ± 2 h whereas MOX agar plates were incubated at 35°C for 48 ± 2 h. Campy-Cefex agar plates were used for the enumeration of *Campylobacter* spp., The agar plates were incubated at 42°C for 48 ± 2 h. Post incubation, typical colonies were counted from each of the countable plates for each sample to determine the CFU/ml value for the sample, and these CFU/ml values were converted to their corresponding $\log_{10}$ CFU/ml values for each organism type.

To determine the presence/absence of *Campylobacter* spp., FDA BAM Chapter 7: Campylobacter modified was used. Briefly, each 50 ml inoculated sample portion was retrieved on the day of testing. The pH of the raw milk was verified to be within the 6-8 range. The raw milk was centrifuged at maximum speed of 12,000 g for 40 minutes. The supernatant was discarded and the pellet (not fat layer) was mixed with 10 ml of 1X Bolton broth. Another 90 ml of Bolton broth were added and samples were incubated at 35°C for 4 h using Campy Pak (BD Difco) to maintain a microaerobic environment. Post incubation at 35 °C, the samples were moved to incubation at 42°C for 48 h. These samples were retrieved at 24 and 48 h to be struck onto Campy Cefex agar plates. These agar plates were incubated micro-aerobically at 42°C for 48 h. The plates were observed for the presence/absence of typical *Campylobacter* colonies; the presence of typical colonies on either set of plates from 24 or 48 h time points indicated positive samples for *Campylobacter* species.

4.4 *Analysis of Uninoculated Samples*

Uninoculated samples were used for the enumeration of indicator organisms (Total Aerobic Plate counts (APC), Psychrotrophic Plate count (PPC), Total Coliforms (TOC), Total Lactic Acid Bacteria (LAB), and Yeasts and Molds (YM) on day 0 and day 14 from each lot of raw milk. The pH values were also determined on Days 0 and 14. For this, a 50 ml portion was stored at 4.4 $\pm$ 1°C for 14 days. For the enumeration of the indicator organisms, the raw milk sample was shaken well and one ml volume of the test sample was diluted as appropriate and plated on 3M^{TM} Total Aerobic Plate Count Petrifilms and incubated at 35 ± 1°C for 48 ± 2 h for APC. Similarly, another set of APC Petrifilm plate were prepared for PPC counts and these plates were incubated at 7 ± 1°C for 10 days. For the enumeration of total coliforms, 3M^{TM} Coliform Petrifilms were used and the plates were incubated at 35 ± 1°C for 24 ± 2 h. For the enumeration of total Lactic Acid Bacteria, 3M^{TM} Lactic Acid Petrifilms were used and the plates were incubated at 35 ± 1°C for 72 ± 2 h. For YM counts,

3M™ Yeast and Mold Petrifilms were used and the plates were incubated at 25 ± 1°C for 7 days. In addition, the pH of the raw milk was also measured on day 0 and day 14.

5.0 RESULTS

The results of the study are presented in the tables and figures below.

The pH values of the raw milk ranged from 6.28 - 7.14 (Table 3). The enumeration of indicators resulting in APC, LAB, YM, and Psychrotrophic counts are also provided in Table 3.

Figure 1. Shows the log-transformed concentrations of all pathogens at both inoculum levels.

Table 4 shows the concentration of cells in CFU/ml when inoculated at Inoculum level I for all four pathogens: *Salmonella enterica* spp., *E. coli* O157:H7, *Campylobacter* spp., and *Listeria monocytogenes* in raw milk at refrigerated storage. The targeted inoculum level was < 10 CFU/ml but the actual inoculum level was determined to range from 22-162 CFU/ml post-inoculation on Day 0.

Table 5 shows the concentration of cells in CFU/ml when inoculated at Inoculum level II for all four pathogens: *Salmonella enterica* spp., *E. coli* O157:H7, *Campylobacter* spp., and *Listeria monocytogenes* in raw milk at refrigerated storage. The targeted inoculum level was ~1000 CFU/ml (3 logs) and the actual inoculum level was determined to range from 600-8,300 CFU/ml post-inoculation on Day 0.

Table 6 shows the concentration of cells in $\log_{10}$ CFU/ml when inoculated at Inoculum level I for all four pathogens: *Salmonella enterica* spp., *E. coli* O157:H7, *Campylobacter* spp., and *Listeria monocytogenes* in raw milk at refrigerated storage.

Table 7 shows the concentration of cells in $\log_{10}$ CFU/ml when inoculated at Inoculum level II for all four pathogens: *Salmonella enterica* spp., *E. coli* O157:H7, *Campylobacter* spp., and *Listeria monocytogenes* in raw milk at refrigerated storage.

Table 8 shows the presence and absence of *Campylobacter* spp. in raw milk during refrigerated storage over 14 days period.

[THE REMAINDER FO THIS PAGE IS INTENTIONALLY LEFT BLANK]

Table 3. pH and Indicator Organisms (CFU/ml) in Raw Milk during Refrigerated Storage

Milk Shipment	Time Point	Milk Lots	pH	APC	Total Lactic Acid Bacteria	Total Coliform	Total YM	Psychrotrophs
Shipment I	Day 0	A	6.91	1,900	70	30	10	30
		B	6.99	1,540	100	10	10	20
		C	6.98	1,490	70	50	10	10
		Average	6.96	1643	80	30	10	20
	Day 14	A	6.93	510	160	10	100,000	57,000,000,000
		B	6.94	820	270	130	90,000	57,000,000,000
		C	6.78	660	360	10	55,000	57,000,000,000
		Average	6.88	663	263	50	81667	57,000,000,000
Shipment II	Day 0	A	7.14	560	460	10	20	75,000,000
		B	7.12	1,700	340	10	20	125,000,000
		C	7.04	800	470	10	20	200,000,000
		Average	7.1	1020	423	10	20	133,333,333
	Day 14	A	6.58	8,000,000	10	3,000	21,000	2,500,000,000
		B	6.34	440,000,000	10	10	310	2,500,000,000
		C	6.28	1,550,000,000	10	10	300	2,500,000,000
		Average	6.4	666,000,000	10	1,007	7,203	2,500,000,000

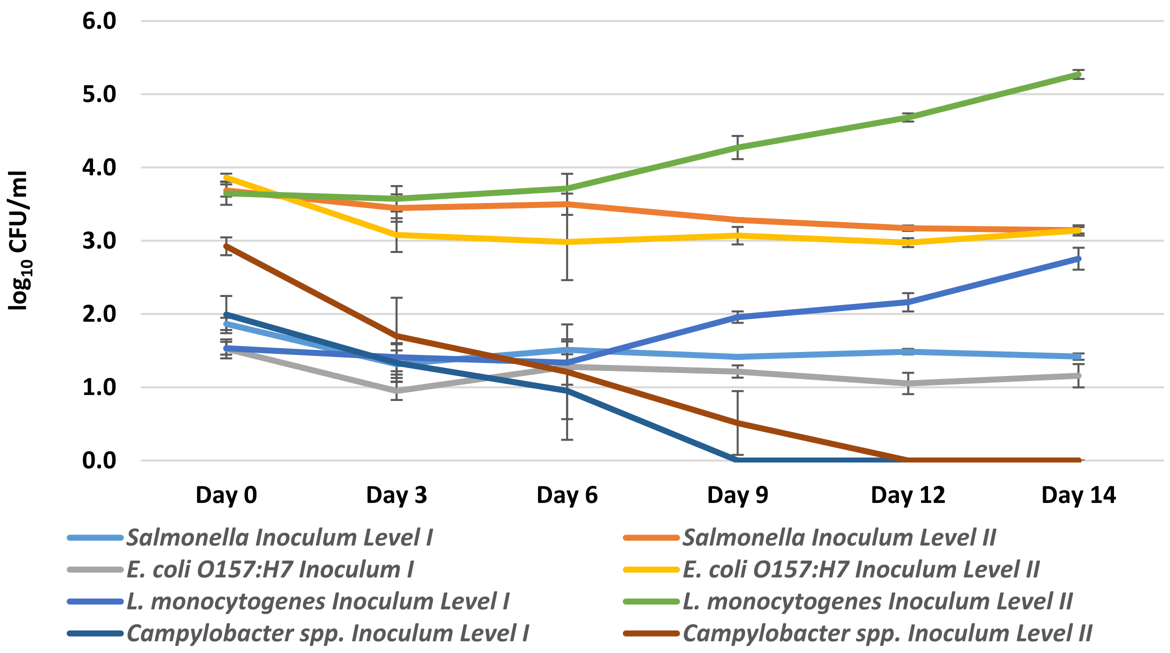

Figure 1. Log Transformed Concentrations of Pathogens at Two Inoculum Levels during Refrigerated Storage

Table 4. Concentration of Cells (CFU/ml) in Raw Milk at Inoculum Level I

Pathogens	Sample Types	Day 0	Day 3	Day 6	Day 9	Day 12	Day 14
Salmonella enterica spp.	Lot A Sample 1	72	22	44	73	132	113
	Lot A Sample 2	86	16	36	35	38	30
	Lot B Sample 1	56	17	37	22	20	11
	Lot B Sample 2	81	20	28	17	42	52
	Lot C Sample 1	66	31	32	18	11	12
	Lot C Sample 2	84	21	22	18	18	14
	Average	74	21	33	31	44	39
	Std. dev	12	5	8	22	45	40
E. coli O157:H7	Lot A Sample 1	33	5	20	16	13	18
	Lot A Sample 2	40	10	10	22	10	22
	Lot B Sample 1	22	10	40	12	8	13
	Lot B Sample 2	25	10	30	17	8	19
	Lot C Sample 1	46	10	20	17	13	10
	Lot C Sample 2	42	10	10	16	19	9
	Average	35	9	22	17	12	15
	Std. dev	10	2	12	3	4	5
L. monocytogenes	Lot A Sample 1	27	33	18	96	179	668
	Lot A Sample 2	38	38	25	82	194	848
	Lot B Sample 1	36	42	26	75	147	436
	Lot B Sample 2	40	18	31	77	156	332
	Lot C Sample 1	26	22	18	106	134	708
	Lot C Sample 2	41	14	16	116	86	584
	Average	35	28	22	92	149	596
	Std. dev	7	11	6	17	38	188
Campylobacter spp.	Lot A Sample 1	43	28	35	<1	<1	<1
	Lot A Sample 2	51	44	42	<1	<1	<1
	Lot B Sample 1	146	10	2	<1	<1	<1
	Lot B Sample 2	162	26	1	<1	<1	<1
	Lot C Sample 1	144	11	20	<1	<1	<1
	Lot C Sample 2	121	28	9	<1	<1	<1
	Average	111.17	24.50	18.17	<1	<1	<1
	Std. dev	51.46	12.65	17.29	.	.	.

Note: The Limit of detection was 1 CFU/ml and samples resulting in no counts are indicated as <1.

Table 5. Concentration of Cells (CFU/ml) in Raw Milk at Inoculum Level II

Pathogens	Sample Types	Day 0	Day 3	Day 6	Day 9	Day 12	Day 14
Salmonella enterica spp.	Lot A Sample 1	5,100	1,740	2,300	1,870	1,420	1,290
	Lot A Sample 2	6,200	1,920	2,000	2,160	1,300	1,420
	Lot B Sample 1	4,200	2,050	4,200	1,900	1,570	1,240
	Lot B Sample 2	4,700	3,700	3,100	1,870	1,460	1,430
	Lot C Sample 1	3,700	4,500	4,700	1,890	1,510	1,360
	Lot C Sample 2	5,700	4,200	3,500	1,860	1,670	1,650
	Average	4,933	3,018	3,300	1,925	1,488	1,398
	Std. dev	931	1,252	1,053	116	127	144
E. coli O157:H7	Lot A Sample 2	7,500	1,370	370	1,070	910	1,350
	Lot B Sample 1	8,200	1,800	400	1,840	1,030	1,540
	Lot B Sample 2	8,300	420	4,700	1,110	1,170	1,780
	Lot C Sample 1	7,300	1,620	4,200	1,040	910	1,170
	Lot C Sample 2	6,700	1,210	450	830	770	1,390
	Average	7,600	1,284	2,024	1,178	958	1,446
	Std. dev	663	534	2,222	386	150	229
L. monocytogenes	Lot A Sample 1	7,900	4,800	9,300	15,400	41,600	241,600
	Lot A Sample 2	5,700	4,700	4,300	16,300	51,200	159,200
	Lot B Sample 1	3,300	5,300	4,700	39,200	49,600	180,800
	Lot B Sample 2	3,000	4,000	5,800	17,400	48,000	176,800
	Lot C Sample 1	4,300	1,800	7,100	16,500	58,800	178,400
	Lot C Sample 2	4,000	3,200	2,400	15,400	42,400	192,800
	Average	4,700	3,967	5,600	20,033	48,600	188,267
	Std. dev	1,830	1,288	2,397	9,420	6,318	28,265
Campylobacter spp.	Lot A Sample 1	600	40	30	<1	<1	<1
	Lot A Sample 2	610	50	12	<1	<1	<1
	Lot B Sample 1	1000	20	70	3	<1	<1
	Lot B Sample 2	1100	240	<1	6	<1	<1
	Lot C Sample 1	1100	10	20	6	<1	<1
	Lot C Sample 2	800	160	37	11	<1	<1
	Average	868.33	86.67	28.17	6.50	<1	<1
	Std. dev	231.55	92.45	24.30	3.32	.	.

Note: The Limit of detection was 1 CFU/ml and samples resulting in no counts are indicated as <1.

Table 6. Log Transformed Cell Concentration (log_{10} CFU/ml) in Raw Milk at Inoculum Level I

Pathogens	Sample Types	Day 0	Day 3	Day 6	Day 9	Day 12	Day 14
Salmonella enterica spp.	Lot A Sample 1	1.86	1.34	1.64	1.86	2.12	2.05
	Lot A Sample 2	1.93	1.20	1.56	1.54	1.58	1.48
	Lot B Sample 1	1.75	1.23	1.57	1.34	1.30	1.04
	Lot B Sample 2	1.91	1.30	1.45	1.23	1.62	1.72
	Lot C Sample 1	1.82	1.49	1.51	1.26	1.04	1.08
	Lot C Sample 2	1.92	1.32	1.34	1.26	1.26	1.15
	Average	1.87	1.32	1.51	1.42	1.49	1.42
	Std. dev	0.07	0.10	0.11	0.25	0.38	0.41
	Reduction/Growth	0.00	-0.55	-0.35	-0.45	-0.38	-0.45
E. coli O157:H7	Lot A Sample 2	1.52	0.70	1.30	1.20	1.11	1.26
	Lot B Sample 1	1.60	1.00	1.00	1.34	1.00	1.34
	Lot B Sample 2	1.34	1.00	1.60	1.08	0.90	1.11
	Lot C Sample 1	1.40	1.00	1.48	1.23	0.90	1.28
	Lot C Sample 2	1.66	1.00	1.30	1.23	1.11	1.00
	Average	1.62	1.00	1.00	1.20	1.28	0.95
	Std. dev	1.52	0.95	1.28	1.22	1.05	1.16
	**Reduction/Growth**	**0.00**	**-0.57**	**-0.24**	**-0.31**	**-0.47**	**-0.37**
L. monocytogenes	Lot A Sample 1	1.43	1.52	1.26	1.98	2.25	2.82
	Lot A Sample 2	1.58	1.58	1.40	1.91	2.29	2.93
	Lot B Sample 1	1.56	1.62	1.41	1.88	2.17	2.64
	Lot B Sample 2	1.60	1.26	1.49	1.89	2.19	2.52
	Lot C Sample 1	1.41	1.34	1.26	2.03	2.13	2.85
	Lot C Sample 2	1.61	1.15	1.20	2.06	1.93	2.77
	Average	1.53	1.41	1.34	1.96	2.16	2.76
	Std. dev	0.09	0.19	0.11	0.08	0.12	0.15
	**Reduction/Growth**	**0.00**	**-0.12**	**-0.20**	**0.43**	**0.63**	**1.22**
Campylobacter spp.	Lot A Sample 1	*1.63*	*1.45*	1.54	0.00	0.00	0.00
	Lot A Sample 2	*1.71*	1.64	1.62	0.00	0.00	0.00
	Lot B Sample 1	*2.16*	1.00	0.30	0.00	0.00	0.00
	Lot B Sample 2	*2.21*	1.41	0.00	0.00	0.00	0.00
	Lot C Sample 1	*2.16*	1.04	1.30	0.00	0.00	0.00
	Lot C Sample 2	*2.08*	1.45	0.95	0.00	0.00	0.00
	Average	1.99	1.33	0.95	0.00	0.00	0.00
	Std. dev	0.25	0.25	0.67	0.00	0.00	0.00
	**Reduction/Growth**	0.00	-0.66	-1.04	-1.99	-1.99	-1.99

Note: The log growth/reduction was calculated by subtracting the mean log_{10} CFU/ml value at a given day from the starting Day 0 concentration: Positive values in green indicate log reduction and negative values in red indicate log growth during refrigerated storage.

Table 7. Log Transformed Cell Concentration (log_{10} CFU/ml) in Raw Milk at Inoculum Level II

Pathogens	Sample Types	Day 0	Day 3	Day 6	Day 9	Day 12	Day 14
Salmonella enterica spp.	Lot A Sample 1	3.71	3.24	3.36	3.27	3.15	3.11
	Lot A Sample 2	3.79	3.28	3.30	3.33	3.11	3.15
	Lot B Sample 1	3.62	3.31	3.62	3.28	3.20	3.09
	Lot B Sample 2	3.67	3.57	3.49	3.27	3.16	3.16
	Lot C Sample 1	3.57	3.65	3.67	3.28	3.18	3.13
	Lot C Sample 2	3.76	3.62	3.54	3.27	3.22	3.22
	Average	3.69	3.45	3.50	3.28	3.17	3.14
	Std. dev	0.08	0.19	0.15	0.03	0.04	0.04
	**Reduction/Growth**	**0.00**	**-0.24**	**-0.19**	**-0.40**	**-0.52**	**-0.54**
E. coli O157:H7	Lot A Sample 2	3.88	3.14	2.57	3.03	2.96	3.13
	Lot B Sample 1	3.91	3.26	2.60	3.26	3.01	3.19
	Lot B Sample 2	3.92	2.62	3.67	3.05	3.07	3.25
	Lot C Sample 1	3.86	3.21	3.62	3.02	2.96	3.07
	Lot C Sample 2	3.83	3.08	2.65	2.92	2.89	3.14
	Average	3.88	3.06	3.02	3.06	2.98	3.16
	Std. dev	0.04	0.25	0.57	0.13	0.07	0.07
	**Reduction/Growth**	**0.00**	**-0.82**	**-0.86**	**-0.82**	**-0.90**	**-0.72**
L. monocytogenes	Lot A Sample 1	3.90	3.68	3.97	4.19	4.62	5.38
	Lot A Sample 2	3.76	3.67	3.63	4.21	4.71	5.20
	Lot B Sample 1	3.52	3.72	3.67	4.59	4.70	5.26
	Lot B Sample 2	3.48	3.60	3.76	4.24	4.68	5.25
	Lot C Sample 1	3.63	3.26	3.85	4.22	4.77	5.25
	Lot C Sample 2	3.60	3.51	3.38	4.19	4.63	5.29
	Average	3.65	3.57	3.71	4.27	4.68	5.27
	Std. dev	0.16	0.17	0.20	0.16	0.06	0.06
	**Reduction/Growth**	**0.00**	**-0.07**	**0.06**	**0.63**	**1.04**	**1.62**
Campylobacter spp.	Lot A Sample 1	2.78	1.60	1.48	0.00	0.00	0.00
	Lot A Sample 2	2.79	1.70	1.08	0.00	0.00	0.00
	Lot B Sample 1	3.00	1.30	1.85	0.48	0.00	0.00
	Lot B Sample 2	3.04	2.38	0.00	0.78	0.00	0.00
	Lot C Sample 1	3.04	1.00	1.30	0.78	0.00	0.00
	Lot C Sample 2	2.90	2.20	1.57	1.04	0.00	0.00
	Average	2.92	1.70	1.21	0.51	0.00	0.00
	Std. dev	0.12	0.52	0.65	0.44	0.00	0.00
	**Reduction/Growth**	0.00	-1.23	-1.71	-2.41	-2.92	-2.92

Note: The log growth/reduction was calculated by subtracting the mean log_{10} CFU/ml value at a given day from the starting Day 0 concentration: Positive values in green indicate log reduction and negative values in red indicate log growth during refrigerated storage.

Table 8. Determination of Presence/Absence of Campylobacter spp. in 50 ml portion Samples During Refrigerated Storage

Campylobacter spp. Inoculum Level	Sample Type	**Day 0**	**Day 3**	**Day 6**	**Day 9**	**Day 12**	**Day 14**
Inoculum Level I	Lot A Sample 1	Positive	Positive	Negative	Negative	Negative	Negative
	Lot A Sample 2	Positive	Negative	Negative	Negative	Negative	Negative
	Lot B Sample 1	Positive	Negative	Negative	Negative	Negative	Negative
	Lot B Sample 2	Positive	Negative	Negative	Negative	Negative	Negative
	Lot C Sample 1	Positive	Negative	Negative	Negative	Negative	Negative
	Lot C Sample 2	Positive	Negative	Negative	Negative	Negative	Negative
Inoculum Level II	Lot A Sample 1	Positive	Positive	Positive	Positive	Positive	Positive
	Lot A Sample 2	Positive	Positive	Positive	Positive	Positive	Positive
	Lot B Sample 1	Positive	Positive	Negative	Positive	Positive	Positive
	Lot B Sample 2	Positive	Positive	Negative	Positive	Positive	Positive
	Lot C Sample 1	Positive	Positive	Negative	Positive	Positive	Positive
	Lot C Sample 2	Positive	Positive	Negative	Positive	Positive	Positive

Note: 50 ml sample portions were inoculated and tested using modified FDA BAM Chapter 7: *Campylobacter* spp.

6.0 CONCLUSION

The results from this study show that the *Salmonella enterica* spp., *E. coli* O157:H7, and *Campylobacter* spp. did not grow in raw milk during the 14 days of refrigerated storage. Similarly, *L. monocytogenes* showed no growth until day 9 and day 6 for the 'Inoculum Level I' and 'Inoculum Level II' samples, respectively. On the other hand, *L. monocytogenes* showed growth starting at day 9 for the 'Inoculum level I' samples and from day 6 for the 'Inoculum level II' samples. For *Campylobacter* spp., multiple, or all the samples inoculated with *Campylobacter* spp. reached below the limit of detection of <1 CFU/ml starting day 6. However, 'Inoculum Level II' samples were still observed to be positive on days 9, 12, and 14 for *Campylobacter* spp. when counts showed <1 CFU/ml, indicating lower detection limits for presence/absence tests.

7.0 FULL REPORT APPROVAL

Approved By:

__________________________ _________________

Sarah Smith/Mark McAfee — Date
Raw Milk Institute (RAWMI)
