# Supplementary material for: Suppression of pathogens in properly refrigerated raw milk
Source: PLoS One. 2023 Dec 12;18(12):e0289249. doi: 10.1371/journal.pone.0289249 (PMC10715650; doi:10.1371/journal.pone.0289249)
Supplement: S2 Appendix — (PDF) [file pone.0289249.s002.pdf]

SRA Risk Analysis Quality Test

Release 1.0

John Lathrop
Willy Roed
Steven Ackerlund
Robert Waller
Terje Aven
Roger Flage
Ghaidaa Hetou
Richard Belzer
Amina Wilkins
Tim Trevan
Ted Yellman
Ted Simon
Irem Dikmen
Samuel Denard
Emma Soane
Tim Smith
Debra Decker
Patricia Larkin
Ronald Dyer

Introduction

- This document presents a number of tests to be used to evaluate the quality of risk analyses supporting risk management decisions. Each test is presented as a challenging question. We title this document Risk Analysis Quality Test, singular, to emphasize that this is a "battery" of tests, to stipulate that all of these tests should be applied, not just any subset. More broadly, we are saying that risk analysis quality is defined by, is tested by, all of these tests, not just any subset.

- All of these tests are consistent with and supported by three iconic documents found on the SRA website: Risk Analysis: Fundamental Principles; Society for Risk Analysis Glossary; and Core Subjects of Risk Analysis.

- That said, these AQTs present emphases that are markedly different than the emphases found in those three documents, because these AQTs were assembled jointly by the authors, focusing on their experiences with pitfalls and shortcomings they have observed in practice with analyses supporting risk management decisions. That is, this RAQT battery is "experienced-pitfall-based."

- Risk analysts are expected to answer each quality question, each AQT, with one of three responses:
  - Yes (then summarize the corresponding analysis quality feature)
  - No (then summarize the implications of that shortfall for risk management decision making and develop a corresponding solution, worded as an "Opportunity To Improve")
  - NA (Not Applicable; realizing that any "NA" could be questioned by any party, so a NA should be accompanied by a justification if it is not obvious).

  Some of the AQTs are multi-part, e.g., A1, in which case each part is to be answered Yes/No/NA. In all cases each response should be in clear summary terms readily understandable by decision makers, including ones who are not experts in risk analysis.

- We have four goals:
  1. To provide a standard procedure and standard "yardstick" with which to define and measure the quality of risk analyses supporting risk management decisions.
  2. To provide "Full Disclosure," not insistence on any ideal analysis quality. That is, in the real world of budgets, schedules, competing interests, and other decision factors, no analysis can score a "Yes" or "NA" on every test here. But risk management decision makers using risk analysis should be made aware of any shortfalls, and the implications of those shortfalls for their decision making.
  3. To consider every discovered shortfall as an "Opportunity To Improve."
  4. To create a culture of analysis quality, where every involved analyst and decision maker is aware of the shortfalls, their implications, and the need to, and ways to, improve the risk analysis

- Many times here we refer to "risk management decision makers," in particular associated with the phrases "should be made aware of," "understandable to" and related phrases. In all such cases, we mean both decision makers who are experts in risk analysis and decision makers who are NOT experts in risk analysis.

- By "analysis," here we mean all of the analysis steps involved in supporting risk management decisions, that is:
  - setting context
  - stakeholder involvement
  - risk identification
  - risk assessment
  - risk communication
  - risk management decision making, and
  - risk governance

- These AQTs are designed to apply to all application areas where risk analyses support risk management decisions. A good list of those application areas can be surmised by simply reviewing 15 of the 16 Specialty Groups listed on the SRA website, all but the Foundational Issues Specialty Group.

- This RAQT battery may seem overly long. We have three responses:
    1. A conscientious review process should be able to complete this review within a reasonable amount of time and effort.
    2. For any particular analysis, a large number of these AQTs will probably have "NA" responses.
    3. These AQTs should all be addressed. It is indefensible to maintain that only some of these AQTs should be addressed since to address all of them would be "too difficult."
- This RAQT battery will be implemented by software that will facilitate responses to each AQT, generate a full results report, and provide a choice among summary reports that can be quickly reviewed for analysis quality control. An announcement will be made on sra.org when the software launches.
- An academic study of risk analysis could more than double the AQTs we list here. Our goal is not to be complete, but to identify the AQTs that most efficiently determine analysis quality in practice.
- It would be impractical to craft these AQTs fully specified at a detailed level for every analysis in every area of application. So these AQTs are crafted very generally, and we depend on reviewers of each analysis to do a responsible job of applying each AQT to the analysis being reviewed.
- This battery of tests will be continually improved over time. Each application will result in additional insights into new questions, reworded questions, examples to include, etc. Changes to this document will be marked by release number.
- The Society for Risk Analysis encourages broad application of this Risk Analysis Quality Test to evaluate risk analyses in support of risk management decisions. The citation to note in resulting reports is: Risk Analysis Quality Test of the Society of Risk Analysis.

Disclaimer

The RAQT is a battery of questions intended to improve the quality of risk analyses done in support of risk management decisions. The RAQT is a work in progress, is provided "as is", and may be revised based on feedback and further evaluation. SRA makes no representations or warranties of any kind and disclaims all responsibility concerning the suitability, inaccuracies, typographical errors, or other potentially harmful components in the RAQT and further disclaims all warranties, express or implied, including but not limited to warranties of merchantability, fitness for a particular purpose and noninfringement. Successful completion of the RAQT should not be used as a definitive statement about the quality of a risk analysis. In no event will SRA be liable for any claim for damages, direct or indirect, incidental, special, punitive, or consequential, however caused and on any theory of liability, whether in contract, tort or otherwise, arising from the use of, or inability to use, or otherwise in connection with the RAQT, or from any action or decision taken as a result of using the RAQT.

Any software implementation of the question battery includes inherent dangers which are present in the use of any software, and the user is solely responsible for determining whether the RAQT software implementation is compatible with the user's equipment or other software installed on the user's equipment. SRA makes no representation or warranties of any kind and disclaims all responsibility concerning any software implementation of the RAQT, extending to such issues as viruses and data privacy.

CONTENTS

Category A. Framing the Analysis and Its Interface With Decision Making 7

AQT A1: Clarity of the goal of the analysis

AQT A2: Clarity of the decisions to be supported

AQT A3: Decision focus of the risk analysis

AQT A4: Adequately diverse set of perspectives consulted in the naming and framing of the problem

AQT A5: Appropriately positioned in the organization chart

AQT A6: Embedding in the decision process

AQT A7: Decision maker focus

AQT A8: Analysis report formats designed to be as helpful as possible to decision makers

AQT A9: Adequate breadth, depth and detail to support the risk management decisions

AQT A10: Societal and stakeholder acceptability

Category B. Capturing the Risk Generating Process (RGP) 8

AQT B1: Comprehensiveness of hazards/events, scenarios, scenarios "Not On the List," implications

AQT B2: Basic Structure of the RGP fully understood and taken into account

AQT B3: Complexity of the RGP fully understood and taken into account

AQT B4: Process for detecting early warnings

AQT B5: Consider possibility of system changes

Category C. Risk Communication 9

AQT C1: Communication integrated into the risk analysis following norms, e.g., ISO 31000, IRGC

AQT C2: Communication adequate between analysts, decision makers and stakeholders

Category D. Stakeholder Involvement 9

AQT D1: Stakeholders identified, consulted and engaged

Category E. Assumptions and Scope Boundary Issues 10

AQT E1: All important assumptions and their implications listed in language clear to decision makers

AQT E2: Evaluation of the risk that an assumption deviates from the RGP

AQT E3: All important scope boundary issues and their implications listed in language clear to DMs

Category F. Proactive Creation of Alternative Courses of Action 10

AQT F1: Process to create alternative courses of action other than what originally considered

Category G. Basis of Knowledge 11

AQT G1: Characterize the basis of each of the key sources of knowledge used in the analysis

AQT G2: Characterize the strength of knowledge in terms of its adequacy to support the decisions

AQT G3: Clearly communicate to decision makers where limitations of knowledge call for risk management strategies that take those limitations into account

AQT G4: Consider the role and importance of surprises and unforeseen events, Black Swans

AQT G5: Consider conflicting opinions between experts

AQT G6: Unconsidered knowledge, i.e., knowledge known to others, outside the analysis group

AQT G7: Consider that some events may have been disregarded as too unlikely, when that is based on assumptions

Category H. Data Limitations, Availability, Collection, Management, Verification, Validation 12

AQT H1: Analyze data limitations and the implications of those limitations

AQT H2: Manage data with a system that logs all data and maintains data QA/QC, documented

AQT H3: Test data for reproducibility

AQT H4: Verify data for internal consistency

AQT H5: Validate data against external reference points

Category I. Analysis Limitations 12

AQT I1: Describe all analysis limitations as they apply to the risk management problem

AQT I2: Verify all calculations, including with sensitivity analyses

AQT I3: Check that all metric levels in the results are supported by metrically valid operations

Category J. Uncertainty: Sources, Characterization, Implications for Risk Management 13

AQT J1: List and characterize all uncertainties in one place, along with their implications

AQT J2: Characterize any aleatory uncertainty in terms decision makers can understand

AQT J3: Characterize the propagation of that aleatory uncertainty into results, in understandable terms

AQT J4: Characterize any data-limitation uncertainty in terms decision makers can understand

AQT J5: Characterize the propagation of that data-limitation uncertainty into results, in understandable terms

AQT J6: Characterize any expert-judgment uncertainty in terms decision makers can understand

AQT J7: Characterize the propagation of that expert-judgment uncertainty into results, in understandable terms

AQT J8: Characterize any expert-disagreement uncertainty in terms decision makers can understand

AQT J9: Characterize the propagation of that expert-disagreement uncertainty into results, in understandable terms

AQT J10: Generate scenarios in a process strongly encouraging “casting a broad enough net”

AQT J11: Generate scenarios in a process that aggressively tests system interactions

AQT J12: Characterize any scenario uncertainty in terms decision makers can understand

AQT J13: Characterize the implications of that scenario uncertainty for risk management decisions in understandable terms

AQT J14: Characterize any model uncertainty in terms decision makers can understand

AQT J15: Characterize the propagation of that model uncertainty into results, in understandable terms

AQT J16: Characterize the combined uncertainty from all uncertainty sources in the results in terms decision makers can understand

AQT J17: Make the implications of that combined uncertainty for risk management decisions clear to decision makers

Category K. Consideration of Alternative Analysis Approaches 15

AQT K1: Consider all plausible analysis approaches, then logically select among them

AQT K2: Make clear to decision makers the implications of choosing an alternative approach

Category L. Robustness and Resilience of Action Strategies 15

AQT L1: Explicitly examine the need for robustness and resilience of action strategies

AQT L2: Test that the recommended risk management strategies include the robustness and resilience called for

Category M. Model and Analysis Validation and Documentation 16

AQT M1: Validate the model and analysis

AQT M2: Document that validation for third party review

Category N. Reporting 16

AQT N1: Define all key terms

AQT N2: Explain and motivate all results without using abstract terms

AQT N3: If results are not as expected, explain

AQT N4: Disclose all possible conflicts of interest

AQT N5: Disclose all funding sources and amounts

Category O. Budget and Schedule Adequacy 16

AQT O1: Test that the budget and schedule are adequate for adequate analysis quality and defensibility

Category A

Framing the Analysis and Its Interface With Decision Making

A1. Clarity of the goal of the analysis.

1. Is the goal of the analysis clear and clearly announced? So that all parties can work toward that same goal without special communication.
2. Is the risk/cost of falling short of that goal described? So that all parties are appropriately motivated to achieve that goal.

Example goals: to assure a safe design, to develop a safe design, to select the best design, to demonstrate the level of safety to others, to defend a proposed action, to evaluate insurance or risk management policies.

A2. Are the decisions to be supported by this risk analysis clearly identified, including clear descriptions of the decision alternatives? Example decisions: go/no-go on a project or action, or decide among actions, strategies or policies. In some cases the goal (A1) is to defend a proposed action. In those cases the decision can be framed as between the proposed action and whatever would happen if the proposed action is not taken.

A3. Is the risk analysis "decision focused"? That is, is the analysis specifically focused on supporting the decision makers in deciding among those decision alternatives?

A4. Are an adequately diverse set of perspectives (i.e., different risk management and stakeholder parties) effectively consulted in the naming and framing of the risk management problem, including scoping?

A5. Is the risk analysis positioned appropriately in the organization chart of the client? Points in the organization chart may range from tactical to strategic, from risk management to management to enterprise management, etc. For example, does the risk analysis deliver results to points in that chart (perhaps several points), such that for each point, it has the appropriate funding, timing and credibility?

A6. Embedding in the Decision Process

- **A6.1** Is the risk analysis fully and effectively engaged with the risk management decision makers? That includes including the decision makers effectively and intimately in problem formulation.
- **A6.2** Does the risk analysis timeline effectively support specific points in decision making?

A7. Decision Maker Focus

- **A7.1** Does the risk analysis give risk management decision makers risk information customized to their perspectives? That is, is the analysis shaped to each risk manager's ability to address the risk, e.g., statutory authority, and to his or her legal requirements?
- **A7.2** Does the risk analysis support risk management decision makers to:
  - » Understand the limitations of the analyses, and the implications of those limitations for their decisions?
  - » Make tradeoffs against "other decision factors"?
  - » Address flaws in the risk management processes?

A8. Are the analysis report formats – numerical, graphical and text – explicitly and deliberately designed to be as helpful as possible to risk management decision makers, in combining the results of the analysis with the "other decision factors" they may face in making their decisions?

A9. Does the risk analysis have an adequate level of breadth, depth and detail to support the risk management decisions being supported?

A10. Are societal and stakeholder acceptability systematically evaluated in:

- **A10.1** The risk management process?
- **A10.2** Any associated recommended risk management actions?

Category B
Capturing the Risk Generating Process (RGP)

B1. Comprehensiveness

- **B.1.1** Is there a structured taxonomy of hazards/events that is evidence of comprehensiveness? Note that "events" can include opportunities, i.e., uncertain events causing benefits.
- **B1.2** Is each scenario spelled out with the causes of change and types of change?
- **B1.3** Are potential hazards/events/scenarios "not on the list" (surprises, unanticipated events, often referred to as Black Swans) explicitly addressed?
- **B1.4** Are the implications of such hazards/events/scenarios for risk management explicitly described?

B2. Is the basic structure of the Risk Generating Process understood and taken into account? For example:

» Is that process linear vs. chaotic vs. complex adaptive?

» Is the basic structure of the mathematics (e.g., linear, quadratic, exponential, etc.) appropriate for that basic structure of the process?

B3. Is the complexity of the Risk Generating Process fully understood and taken into account in the analysis methods? This can be tested by listing all the important (for the resulting risk) causal and associative links in the RGP, then demonstrating that each of those links is accounted for in the analysis. This need not be as burdensome as it may sound, if the causal and associative links are intelligently selected.

B4. If the context calls for detecting early warnings, is there a process used for that detection? Those early warnings include of potential surprising risk aspects, more broadly than concrete events.

B5. Is the possibility of system changes fully considered and recognized? As part of that: are adequate mechanisms in place to detect those changes?

Category C
Risk Communication

C1. Is communication integrated into the risk analysis following established norms, e.g., using all aspects of:

» The ISO 31000 methodology: e.g., Establishing the context, Risk Assessment (Identification, Analysis, Evaluation), Risk Treatment?

» The International Risk Governance Council (IRGC) methodology: e.g., Pre-Assessment, Management, Appraisal, Characterization & Evaluation? Key: Categorizing the knowledge about the risk, and so related to Category G, Basis of Knowledge.

C2. Have all considerations for effective risk communication been applied to assure adequacy of risk communication between analysts and decision makers?

» Analysts and other stakeholders?

» Decision makers and stakeholders?

In all three cases, "adequate" means both parties agree the communication is adequate.

Category D
Stakeholder Involvement

D1. Are all stakeholders systematically and effectively identified, consulted and engaged, in such a way that all stakeholders would agree that they were effectively consulted and engaged?

That extends to:

» Considering their perceptions and concerns;

» Involvement in the naming, framing, and scoping of the risk management problem;

» Involvement in the risk management decision process;

» Involvement in the risk management implementation process.

Category E

Assumptions and Scope Boundary Issues

E1. Are all important assumptions, and the implications of each such assumption for risk management, listed systematically in language clear to risk management decision makers?

Example: A major model assumed that a critical resource constraint did not apply, as a way to avoid a large analysis burden. That assumption significantly distorted its risk ranking of alternative threats. That distortion was not made clear to decision makers.

The issue addressed in the above AQT has a risk variant. For clarity, we place that risk variant here in a separate AQT:

E2. Each significant assumption may include a risk that that assumption deviates from the actual Risk Generating Process in such a way that the consequences and implications of that assumption are important. For each significant assumption, has that risk been evaluated and has that risk and its possible consequences and implications been made clear to the risk management decision makers?

E3. Are all important scope boundary issues, and the implications of each scope boundary issue for risk management, been listed systematically in language clear to risk management decision makers? Some scope boundary issues may be best addressed in terms of associated assumptions. This AQT is included to highlight scope boundary issues as distinct from assumptions.

Example: A major model limited the scope of consequences considered, as a way to avoid a large analysis burden. That scope decision significantly distorted its risk ranking of alternative threats. That distortion was not made clear to decision makers.

Category F

Proactive Creation of Alternative Courses of Action

F1. Are alternative courses of action systematically generated through a process of proactive, goal-focused creation? In some cases, an analysis to evaluate a course of action to address a situation focuses on only one “alternative” course of action, or a small set of alternatives that has been defined by some unexamined process or a process external to the analysis. A common wisdom in decision analysis is that often the best way to address a situation is to focus on creating alternatives other than the one or few considered. This AQT is designed to promote a process of examining the set of alternatives considered to see if one or more better alternatives can be developed. Of particular concern: Cases where the uncertainty is such that more robust and/or resilient alternatives should be developed, and cases where action-reaction spirals among different parties may lead to unintended consequences.

Category G
Basis of Knowledge

G1. Is the basis of knowledge characterized? For example: Which inputs are empirically "objective," which inputs are Subject Matter Expert (SME) elicitation, which inputs are based on testing, which inputs are based on modeling, which knowledge is based on argumentation and reasoning, which aspects are treated with assumptions, which analyses are broadly accepted, which analyses are one of two or more analyses that are considered acceptable, which analyses are novel and not widely accepted? This characterization of the basis of knowledge may seem impossibly involved in the general case, but for any particular analysis it is quite feasible and of course should be spelled out.

G2. Is the strength of knowledge characterized in terms of its adequacy to support the risk management decisions to be supported? This AQT addresses the issue: Contexts with limited factual knowledge call for risk management recommendations that take those limitations into account.

G3. In cases where limitations of knowledge call for risk management strategies that take those limitations into account, has that been communicated to risk management decision makers in language they can understand and apply?

G4. Is the role and importance of potential surprises and unforeseen events (often referred to as Black Swans) considered? Another description of those: Events and scenarios "not on your list." Some risk management contexts have inconsequential or extremely improbable Black Swans as the phenomena are well understood and the uncertainties are small. In other contexts, e.g., terrorism, Black Swans can be a driving consideration, since terrorists may deliberately design attacks that are "not on the defender's list," Black Swans to the defender. This is a central concern and as such is also touched on in two other categories of this battery:

- Category B: Comprehensiveness of the list of hazards/events;
- Category L: Robustness and Resilience of Action Strategies.

G5. Are conflicting opinions between experts considered as a source of uncertainty and reported to decision makers? This is re-visited in Category J on uncertainty.

G6. Has there been explicit consideration of the possibility of unconsidered knowledge (i.e., knowledge that others have, outside of the analysis group)? That is, have special measures been implemented to check for this type of knowledge (for example, the use of an independent review of the analysis)?

G7. Has there been explicit consideration of the possibility that some events have been disregarded because of very low probabilities, although those probabilities are based on critical assumptions? That is, have special measures been implemented to check for this type of event (for example, signals and warnings concerning the existing knowledge basis)?

Category H

Data Limitations, Availability, Collection, Management, Verification, Validation

H1. Are data limitations systematically analyzed, in particular the implications of those limitations for risk management, then any implications reported to risk managers in language they can understand and apply? Examples of those limitations: Availability and aspects of data collection.

H2. Are the data managed with an adequate data management system that assures each piece of data is accurately logged, and that appropriate levels of QA/QC are maintained, including the ability to demonstrate that adequate level of QA/QC to a third party?

H3. Are the data tested for reproducibility?

H4. Are the data verified for internal consistency?

H5. Where possible, are the data validated against external points of reference? That is, are external points of reference sought, then are the data checked for consistency with those external points?

Category I

Analysis Limitations

I1. Are all analysis limitations, as they apply to the risk management problem, clearly described? That is, are the limitations of the set of calculations of the analysis, including modeling, explicitly examined, in particular as they apply to the overall risk management situation at hand? This is as opposed to other limitations covered in two other categories of this battery:

» Category G: Basis of Knowledge
» Category H: Data Limitations

Notice the overall theme spanning Categories G, H and I: Any risk analysis is subject to limitations in knowledge, data and analyses. Even in the best of cases, those limitations are typically unavoidable. What is important here is that those limitations, and the implications of those limitations, be examined and clearly explained to the risk management decision makers.

I2. Have all calculations in the analysis been verified? That may include extensive sensitivity analyses.

I3. Are all metric levels in results (i.e., nominal, ordinal, interval, ratio) supported by metrically valid operations beginning with the data? For example, if the results include bar charts or other formats that present ratio-scale data (whether or not the analysts intended a ratio-scale presentation), are those results ratio-scale invariant to metric-allowed variations of the source data? For a specific example from experience: A major model elicited ordinal judgments of probability, then multiplied pairs of those judgments and summed those products into results numbers, presented in scatterplots and bar charts. An analysis with alternative transformations of the original data, shifted by transforms allowable for ordinal metrics, resulted in rank reversals in the bar charts. So in that case the results were not even valid as ordinal metrics.

Category J

Uncertainty: Sources, Characterization, Implications for Risk Management

Uncertainty is of course central to any risk analysis, and touches on fundamental issues. In most risk analyses, uncertainty characterization, quantification, calculation, communication, and understanding of how it relates to decision making are central, and subject to shortfalls. And so we organize this category differently than the other categories. We start with an AQT that asks whether or not all of the relevant uncertainties are listed and characterized in one place, then we break those uncertainties down into six categories of uncertainty sources, and for each category we ask AQTs specific to that category. The result is 17 AQTs, which may seem overly thorough, but is commensurate with the importance of these issues. Several different taxonomies of uncertainty sources could be considered. We find this taxonomy to be most useful.

J1. Are all of the significant uncertainties listed in one place, and characterized there, and their implications for decisions described there, in terms risk management decision makers can understand? Do those characterizations provide clear answers on the following key questions: What is uncertain? Who is uncertain? What are the main sources of the uncertainties? How are the uncertainties represented or expressed?

The rest of this category goes into more detail, but it is important that the decision makers get an overview of the uncertainties, as called for here in J1. The uncertainty sources addressed in the following describe the strength of the knowledge supporting the risk characterizations, and so provide a different perspective to the basis of knowledge issues covered in Category G.

Six Uncertainty Sources:

Uncertainty Native to Data (Variation): The part of the uncertainty that is inherent in the set of collectable data, such that, independent of data sampling, there is unavoidable uncertainty in the sampled data. This is sometimes referred to as "aleatory uncertainty." That uncertainty is about variation, and is typically represented using probability models.

J2. Is that aleatory uncertainty characterized in terms risk management decision makers can understand?

J3. Is the propagation of that aleatory uncertainty into results uncertainty characterized in terms risk management decision makers can understand? That propagation should often be analyzed with extensive sensitivity analysis.

Uncertainty Due to Limitations of Data Collection: That is, practical considerations of time and budget typically limit data collection to less than the amount called for for ideal risk management. This type of uncertainty is uncertainty that could be reduced with further time, cost and effort in data collection, provided relevant data are available or can be made available.

J4. Is that data-limitation uncertainty characterized in terms risk management decision makers can understand?

J5. Is the propagation of that data-limitation uncertainty into results uncertainty characterized in terms risk management decision makers can understand? That propagation should often be analyzed with extensive sensitivity analysis.

Uncertainty Arising From Expert Judgment: In cases where the analysis requires expert judgment, even when there is no disagreement among experts (as in the next section), that expert judgement involves uncertainty that can be very significant and very challenging to characterize.

J6. Is that expert-judgment uncertainty characterized in terms risk management decision makers can understand?

J7. Is the propagation of that expert-judgment uncertainty into results uncertainty characterized in terms risk management decision makers can understand? That propagation should often be analyzed with extensive sensitivity analysis.

Uncertainty Arising From Disagreement Among Experts: In cases where the analysis requires expert judgment, special cases arise when different experts provide conflicting judgments. Those are special cases of the expert-judgment uncertainty just described. In fact, the same underlying challenges of uncertainty characterization apply, though this disagreement case is separated out because in this case that uncertainty is typically larger and more clear to non-expert observers.

J8. Is that expert-disagreement uncertainty characterized in terms risk management decision makers can understand?

J9. Is the propagation of that expert-disagreement uncertainty into results uncertainty characterized in terms risk management decision makers can understand? That propagation should often be analyzed with extensive sensitivity analysis.

Uncertainty Captured by Scenarios: This part of the uncertainty includes failure mode scenarios and alternative model-run scenarios. It calls for different AQTs than with the other sources:

J10. Are the scenarios generated in a process that strongly encourages “casting a wide net” to encompass as wide a range of scenarios as called for to capture the uncertainties? That includes “Red Team” processes as commonly understood.

J11. Are the scenarios generated in a process that aggressively tests system interactions?

J12. Is that scenario uncertainty characterized in terms risk management decision makers can understand?

J13. Are the implications of that scenario uncertainty for risk management decisions characterized in terms risk management decision makers can understand?

Model Uncertainty: The part of the uncertainty that is due to the fact that the model used as a basis for the analysis may not fully capture the actual risk generating process. We include in this uncertainty:

- » The uncertainty that arises from disagreement as to which of conflicting models best applies; and
- » Concerns about the model becoming inapplicable after a major disruptive event, e.g., applying to managing the risk of a second major terrorist attack after a first major terrorist attack.

J14. Is that model uncertainty characterized in terms risk management decision makers can understand?

J15. Are the implications of that model uncertainty for uncertainty in the results characterized in terms risk management decision makers can understand? Those implications should often be analyzed with extensive sensitivity analysis.

The above fourteen AQTs (J2-J15) cover six basic sources of uncertainty in risk analysis. But what matters for risk management is the combined results of those six sources for the combined uncertainty in the results. Which leads to the next AQTs:

J16. Are the six sources of uncertainty just discussed combined into a representation of the combined uncertainty in the results, in terms understandable to risk management decision makers? That representation should often be developed with extensive sensitivity analysis, in particular, sensitivity analyses designed to characterize the likelihood that recommended alternatives may turn out to perform less well than other alternatives. Of particular concern: Unsupported precision in results, and uncertainty bars lacking explanation of confidence levels.

J17. Are the implications of that combined uncertainty for risk management decisions made clear to the risk management decision makers, in terms they can understand and apply in their decisions? Of particular concern: Cases where uncertainty is such that analysis should support decision makers in comparing more robust and/or resilient alternatives against alternatives that depend on particular resolutions of uncertainty to perform relatively well.

Category K

Consideration of Alternative Analysis Approaches

K1. In some cases more than one analysis approach could be applied. Are all plausible alternative analysis approaches considered? Then was the adopted analysis approach selected in a logical process?

K2. Are the implications for risk management of choosing an alternative analysis made clear to risk management decision makers?

Category L

Robustness and Resilience of Action Strategies

L1. Is the need for robustness and resilience of action strategies explicitly examined? In this context by robustness we mean the ability of a system to perform well, without adaptation, when impacted by an attack, accident, or other event. By resilience we mean the ability of a system to respond well or adapt well to an attack, accident, or other event. In both cases, "event" includes any change, disturbance, stressor, etc., both anticipated and unanticipated events. This AQT is crucial, and directly relates to Categories B (Scenarios Not On the List, central reasons for robustness and resilience), F (Proactive Creation of Alternative Courses of Action), and I (Analysis Limitations). At base, here, is the recognition that in many areas, a risk analysis cannot confidently take into consideration all scenarios that could happen. From that it follows that, unless the need for robustness and resilience is explicitly examined, the results of the analysis can fall importantly short of adequately supporting risk management decisions.

L2. Do the recommended risk management strategies that follow from the risk analysis include the robustness and resilience called for by the situation? This AQT follows naturally from the one before, and is based on the analysis-limitation logic presented there.

Category M

Model and Analysis Validation and Documentation

M1. Is the model and analysis fully validated, by normal standards of validation in the area of practice that applies?

M2. Is the model, analysis, and validation fully documented, so that a third party review can determine the validity of the model?

Category N

Reporting

N1. Are key terms defined?

N2. Are the results explained and motivated without using abstract terms?

N3. Are the results as expected? If not, is it explained why?

N4. Are all possible conflicts of interest fully disclosed?

N5. Are all funding sources and amounts fully disclosed?

Category O

Budget and Schedule Adequacy

O1. Is the budget and schedule adequate to support the risk analysis at an appropriate level of quality and defensibility? Typically a case can be made for an improved analysis with a larger budget and longer schedule. In the real world there is always a trade-off between analysis quality (as defined by these AQTs), budget and schedule. But this AQT is targeted to situations where a convincing case can be made that the analysis is too restricted by budget and/or schedule to do an adequate job of supporting the risk management decisions at hand.
