# Supplementary material for: Suppression of pathogens in properly refrigerated raw milk
Source: PLoS One. 2023 Dec 12;18(12):e0289249. doi: 10.1371/journal.pone.0289249 (PMC10715650; doi:10.1371/journal.pone.0289249)
Supplement: S1 Table — (DOCX) [file pone.0289249.s004.docx]

**Table S1.** Summary of predictive microbiology studies in raw milk.

| **Reference** | **Initial Inoculum Density** (log cfu/mL) | **Single Strains or Cocktails** (pathogens or non-pathogens) | **Test Matrix**  (culture broth, raw or heated milk) | **Refrigeration Temperatures** (degrees C) | **Abuse Temperatures** (degrees C) | **Reported Growth or Decline at Refrigeration Temperatures** |
| --- | --- | --- | --- | --- | --- | --- |
| Doyle & Roman (1982) | ~10^7^ | Eight *C. jejuni* strains surviving | Raw milk, sterile milk (heated 121° 15 min), brucella broth | 4° | Not tested | No growth; Decline rate at 4°C raw>sterile>broth (Fig. 2); No statistical analysis or curve fitting |
| Northolt et al., 1988; FDA/FSIS, 2003, App. 8^1^ | 10^2.5^ | Cocktail of four strains *L. monocytogenes* | Raw,  pasteurized  milks | 4°,  7°,  4° | Not tested | Exponential growth rates extrapolated to 5° 0.085 (raw) 0.173 (raw) 0.407 (pasteurized) |
| Wang et al. (1997) | 10^3^,  10^6^ | Cocktail of five *E. coli* O157:H7 strains | Raw and pasteurized milks | 5°,  8°, | 15°  22° | No growth at 5°;  Significantly slower growth raw at higher temperatures (p<0.01; Fig. 1); No curve fitting |
| Giacometti et al. (2012a) | 10^2^ | Cocktails of three strains for each:  *C. jejuni,*  *E. coli* O157:H7,  *L. monocytogenes*,  *S. typhimurium* | Raw milk | 4°C as ‘best-case’ scenario | ‘worst-case’ abuse scenario  7°C (for 5 hr)  11°C (for 22.5 hr)  30°C (for 0.5 hr)  12°C (for 68 hr) | No growth at 4°C for *C. jejuni, E. coli* O157:H7, *S. typhimurium*  *L. monocytogenes* increased at 4°C from 2.2 to 2.6 log CFU/mL |
| Castro et al. (2017) | 10^0^,  10^1^,  10^2^ | *L. monocytogenes* | Raw milk | 6°,  8°,  10° | Not tested | *L. monocytogenes* increased at 6°C from:  2 CFU/mL to 2-3 log CFU/mL by 14 days;  20 CFU/mL to 3.5-4.5 log CFU/mL by 14 days;  200 CFU/mL to 4 log CFU/mL by 14 days;  No curve fitting |
| Leclair et al. (2019) | 10^2^,  10^6^ | *E. coli* O157:H7,  *L. monocytogenes* | Raw milk | 4°,  8° | 15°,  22° | Estimated Marginal Means reported, not rates by pathogen and inoculum level;  Both pathogens decline close to baseline levels at lower inoculum level |
| Jaakkonen et al. (2020) | ~10^5^ | 12 single *C. jejuni* strains | Retail organic raw milk | 4° | Not tested | No growth; Decline of mean counts reported by strain over 6 days in Supplemental Table A (with standard deviations, standard errors of mean, 95% confidence); No curve fitting |

^1^Maximum specific growth rates estimated by FDA/FSIS (2003) for data of Northolt et al. (1988) though curve fitting was not conducted in the original study of Northolt and colleagues.
