# Supplementary material for: Suppression of pathogens in properly refrigerated raw milk
Source: PLoS One. 2023 Dec 12;18(12):e0289249. doi: 10.1371/journal.pone.0289249 (PMC10715650; doi:10.1371/journal.pone.0289249)
Supplement: Attachment — Submitted filename: ResponseToEditorReviewers_2June2023.docx [file pone.0289249.s005.docx]

Dear Editor and Reviewers,

Thank you for reviewing our manuscript and considering it for publication in PLOS ONE.

We organize your comments and our responses in three sections:

A. The Editor’s comments;
B. The Reviewers’ Responses to Questions; and
C. The Reviewers Questions and Author Responses.

For Section C, each Reviewer comment is listed separately, followed by Author Responses indented below and marked by italicized text for clarity.

# A. Responses to Editor Comments

1.	We provide a revised manuscript that now conforms to PLOS ONE’s style requirements.

2.	We request the following text replace the original text in our Competing Interests section:

M.E.C. serves as an unpaid advisor on the Raw Milk Institute advisory board and has provided paid consulting services to the Raw Milk Institute and expert testimony in several court cases regarding the microbial ecology and assessment of benefits and risks for microbial pathogens in raw milk and other foods and feeds. This does not alter our adherence to PLOS ONE policies on sharing data and materials. There are no restrictions on use of the minimal dataset provided as Appendix 3 in the Supporting Information section. Other authors have no competing interests to declare.

3.	We now provide under the Supporting Information section both the minimal dataset (Excel file with raw data necessary to replicate our analysis) and the full report from the contracting laboratory who conducted the experiments for the pilot study (including metadata, methods, and experimental data generated in the laboratory). Thank you to the editorial staff for updating our Data Availability statement for our manuscript with this information.

4.	We apologize that our zip file submitted with our original manuscript included versions of the two figures in MS Word with captions, as well as versions of the figures (tif and png formats) without the captions. We now provide both figures with captions in png format. Please advise us if additional detail or formats are needed.

5.	We addressed the study design question from one reviewer and reorganized and greatly shortened the Discussion section as per the comments of that reviewer and the Editor. Detailed responses to Reviewers are provided in Section C below.

# B. Reviewer's Responses to Questions

1. Is the manuscript technically sound, and do the data support the conclusions?

The manuscript must describe a technically sound piece of scientific research with data that supports the conclusions. Experiments must have been conducted rigorously, with appropriate controls, replication, and sample sizes. The conclusions must be drawn appropriately based on the data presented.

Reviewer #1: Partly

Reviewer #2: Yes

2. Has the statistical analysis been performed appropriately and rigorously? Both YES

3. Have the authors made all data underlying the findings in their manuscript fully available? Both YES

The [PLOS Data policy](http://www.plosone.org/static/policies.action#sharing) requires authors to make all data underlying the findings described in their manuscript fully available without restriction, with rare exception (please refer to the Data Availability Statement in the manuscript PDF file). The data should be provided as part of the manuscript or its supporting information, or deposited to a public repository. For example, in addition to summary statistics, the data points behind means, medians and variance measures should be available. If there are restrictions on publicly sharing data—e.g. participant privacy or use of data from a third party—those must be specified.

4. Is the manuscript presented in an intelligible fashion and written in standard English? Both YES

# C. Reviewers' Comments and Authors’ Responses:

**Reviewer #1**: The author studied the growth curves of several pathogenic bacteria in raw milk for direct drinking, and compared them with relevant public reports, providing some support for the change of the growth kinetics of pathogenic bacteria in raw milk. However, there are some defects in the experimental design, such as the number of pathogenic bacteria accessed is only two levels of control, the sampling interval in the early stage of the test is too wide, and the sampling test is only conducted on the 0, 3, and 6 days, which may have a significant impact on the test results.

***Response****: Thank you for your comments. We respond below to your concerns about the study design. We believe that the experimental design of our small pilot study is appropriate for trend analysis and ANOVA. Further, the pilot study design is comparable to that in a manuscript published in this journal that we cited (Jaakkonen et al., 2020). Figure 2 of that paper illustrates a single level of pathogen inoculation at ~10^5^ cfu/mL (we included two inoculation levels) and data on decline at 6 time points after inoculation into raw milk. It is true that Jaakkonen and colleagues sampled daily for 6 days; the pilot study sampling was less frequent, but 6 sampling points were spread over 14 days (days 0, 3, 6, 9, 12, 14).

We note that the distance between sampling points does not influence the statistical analysis by the non-parametric Mann-Kendall test. Also, we report that trends were statistically significant by both tests, and supporting tables and figures for the 48 time series curves generated in the pilot study were provided. Further, decline over 6 sampling points at proper refrigeration temperature is consistent for both the Jaakkonen study and the pilot study. Daily sampling for the Jaakkonen study shows no peaks of growth at days 1, 2, 4, and 5, days that were not sampled in the pilot study. We agree with Reviewer 1 that including more sampling points in a follow-up study is important for parameter estimation, and this concern is included in the future research needs paragraph of our Discussion section.*

**Reviewer #1**: It is not a new discovery that L.monocytogenes will continue to grow during low-temperature storage.

***Response****: Thank you for your comment. It certainly was not our intent to claim a new discovery about L. monocytogenes growth in long term low temperature storage. We revised the Discussion section to emphasize the results for the 3 pathogens associated with all but one US raw milk outbreak in the past 16 years, and de-emphasized discussion for listeriosis.*

**Reviewer #1**: In addition, in the discussion part, the author questioned the role of risk assessment in the formulation of regulations and standards with a large space, which has certain limitations.

***Response****: Thank you for your comment. It was not our intent to question the roles of risk assessment and development of regulations and standards. Our intent was to build on statistical evidence for trends of pathogen growth at proper refrigeration temperatures documented in the pilot study in order to motivate future studies designed specifically for estimation of key microbial risk data gaps, robust parameters for growth and decline.

Also, it is unclear to us if this comment from Reviewer 1 reflects an objection to the wide span of time for the documentation that we reference on microbial risk or a gap that neglects relevant studies. We believe that we provided appropriate references about risk assessment. Our referencing does span multiple decades (11 studies, published in 1999, 2003, 2009, 2011, 2014, 2015, 2017, 2018, 2019, 2021, and 2022), and we are not aware of any relevant risk studies that we did not cite.

We strongly believe that it is important for us to cite the first milestone international consensus document on microbial risk initially ratified by Codex Alimentarius Commission member countries in 1999 (CAC, 1999). For reference, we note that Google Scholar documented 320 citations of the CAC document (as of 11 May 2023) for this historic international agreement about principles and guidelines for microbiological risk assessment completed very early in the evolution of the field. Two of the authors have published in this field over these decades, representing both historic work as well as recent advances in microbial risk and its interconnections with predictive microbiology studies. The work that motivated the pilot study design (Coleman et al., 2003a,b) was relevant to cite in the manuscript, and later risk work from recent years (2009-2022) also had relevance to the interpretation of the pilot study and the needs for future risk research.*

*We also referenced a very recent document with broad international support, the Society for Risk Analysis (SRA) document on risk analysis quality (RAQT, 2021) that one author (MEC) applied to QMRAs for an SRA conference workshop in 2022. Note that we provide the RAQT in full for PLOS ONE readers in the Supporting Information section (S2 Appendix).

We agree with the reviewer that acknowledging the limitations of publications that span decades is appropriate, and we anticipate that the explanations provided herein about the body of literature cited are clear and cogent.*

**Reviewer #1**: The test results can not directly prove that the risk assessment has been out of control.

***Response****: Thank you for your comment. It is unclear to us what section of the manuscript is causing the concern raised by Reviewer 1. We state in our Conclusion section that results from the pilot study in raw milk ‘were consistent with previous studies demonstrating suppression of growth of major bacterial pathogens at proper refrigeration temperatures.’ The second and final sentence of the Conclusion section states the following: ‘Future research is needed to expand the results of the small pilot study on pathogen suppression in raw milks to address risk analysis more holistically, structuring and simulating tradeoffs between benefits and risks of raw and pasteurized milks.’ Thus, from our perspective, the Conclusion sections states that our results are consistent with a body of published studies, and that risk analysis could be improved by incorporating additional scientific data in place of invalid assumptions.

In our revised Discussion section, the three subtitles below frame our application of the results of the pilot study to published literature and reports:
1) ‘Need for reliable data to replace invalid assumptions for robust risk analysis’;
2) ‘Need for evaluation of QMRAs relative to international guidance and quality criteria’; and
3) ‘Need for transparency about scientific evidence falsifying prior assumptions’.
In these subsections, we state that the body of evidence is inconsistent with assumptions in two QMRAs, cite other studies updating the QMRAs, and note that risk analysis quality could be further improved by updating the QMRAs and replacing invalid assumptions with experimental data.

We also inserted a new Table 2 in the Discussion section that is intended to improve clarity about the CAC guidelines and their importance in QMRA processes. CAC Guidelines 10 and 11 indicate an expectation that risk assessments will require updating and reassessment as science advances. We hope that our re-organization of the Discussion section alleviates this concern of Reviewer 1.*

**Reviewer #1**: In this experiment, Salmonella has been kept at a high level. Although Campylobacter has been inactivated at the end of the shelf life, it is not excluded that consumers can contact products that pollute high-level Campylobacter at the early stage of the shelf life,

***Response****: Thank you for your comment. We agree with the interpretation of our results provided by Reviewer 1. It is also clear that results from predictive microbiology experiments could be applied to risk assessment. However, we acknowledge the limitations of the pilot study and the need for additional research to estimate parameters for updating risk assessments in the future. We intentionally limited our Discussion of our results as contributing to a coherent body of evidence that is inconsistent with assumptions of past QMRAs in the Discussion section. Future QMRAs could address the concerns voiced here by Reviewer 1.*

**Reviewer #1**: It is not allowed to exaggerate the safety of direct edible raw milk and belittle the role of pasteurization in milk risk prevention and control without direct evidence.

***Response****: Thank you for your comment. We are uncertain what section of the manuscript is causing the concerns raised here by Reviewer 1. We have taken care to provide relevant references to our statements about the scientific evidence for both raw and pasteurized milks, risk analyses, and a systematic review on milks.

In the Introduction, we state and reference the following:
‘Evidence exists that humans have consumed ruminant milks for millennia (Warinner et al., 2014), well before milk pasteurization became common in the 20^th^ century.’ Further in this section: ‘Notably, the US Food and Drug Administration (FDA) and the Food Safety and Inspection Service (FSIS) jointly reported that both raw and pasteurized milks were high risk foods for severe listeriosis (FDA/FSIS, 2003), and a recent systematic review reported that severe listeriosis risks were significantly higher for pasteurized than raw milks (Sebastianski et al., 2022)’.

Previously in the same paragraph, we cite Cornell University Emeritus Professor Rodney Dietert and colleagues (2022) who published an evidence map on benefits and risks of bovine milks. The following section of the Introduction begins with a statement that reliable and statistically rigorous data are needed for raw milk producers, regulators, and consumers to inform their decisions. Our intention is to cite evidence for risks associated with raw and pasteurized milks, not to exaggerate or compare risks without additional evidence.

In the Discussion, we modified the statement listing four ‘unstated and unsupported assumptions’ about milk (lines 367-371 in the manuscript revision with all changes accepted), providing context for the source in the Society for Risk Analysis workshop, referencing a manuscript in preparation. As stated above, our intent is to support our statements with published studies that provide the scientific evidence that could be applied in risk analysis. We also point out that we did not estimate risk or safety in the pilot study. We are recommending in the Discussion the need for updating QMRAs with scientific evidence to replace incorrect and invalid assumptions, consistent with CAC guidance. The revised Discussion section (lines 380-381 in the manuscript revision, with all changes accepted) includes this summary statement to provide more context: ‘We also acknowledge that no food is risk free, and benefits and risks could and perhaps should be characterized for all foods.’

In addition, we inserted the following text to the Discussion section (lines 358-361 in the manuscript revision, with all changes accepted) that is relevant to comments made by both reviewers: ‘SRA leaders seek to encourage others to apply the SRA RAQT in both review of completed QMRAs for other foods and water, as well as in planning for future risk analysis projects, with the goal of developing a culture of full disclosure and quality analysis.’ We hope that these explanations and expansions alleviate this concern of Reviewer 1.*

**Reviewer #1**: Before considering acceptance, the author should reorganize the discussion section and delete a lot of speculative language.

***Response****: Thank you for your comment. We appreciate the opportunity to reorganize the Discussion section. However, it is unclear what text concerns Reviewer 1 as speculative. We considered the possibility that Reviewer 1 regarded our use of the word ‘likely’ as speculation. We deleted three instances of our use of the word ‘likely’ for this reason, and deleted or substituted its use in the revised Discussion section. We substituted ‘attributable in part’ for ‘likely attributable’ (line 296 in the manuscript revision, with all changes accepted) and ‘evidence is consistent with’ for ‘likely due to’ (line 378 as prior).

We reorganized the Discussion section as follows: i) framed the subsections under 3 subheadings listed above; ii) added Table 2 for relevant CAC guidelines that reduced the number of words describing their application; iii) removed ~500 words from our original submission; and iv) added ~100 words for clarification.

Some deletions and simplifications were made in the discussion of recent epidemiologic evidence from a US CDC dataset including milk outbreaks. Supplemental Table 2 was deleted as a potential distraction from predictive microbiology work and its future applications to risk analysis.*

**Reviewer #2**: Review of manuscript PONE-D-23-04758 “Suppression of Pathogens in Properly Refrigerated Raw Milk”
Summary
The authors address the issue of conflicts in the literature regarding the growth of Foodborne pathogens in raw milk (Campylobacter, E. coli O157:H7, Listeria monocytogenes, or Salmonella). The introduction provides a review of recent findings concerning both foodborne illnesses incidence associated with the above the fore mentioned bacteria from raw and heat-treated milk. They also discuss reports in the literature referring to pathogens inoculated in raw milk having slower growth rates in raw milk than pasteurized milk due to the competing organisms and biologically active components present. The authors commissioned a pilot study through a contract lab to provide further information on the previously mentioned pathogens growth in raw milk.

General Comments
The manuscript is well written.
The pilot study was well designed and used relevant pathogen inoculation numbers.
The statistics were thorough and the data robust.

Specific Comments
The authors make valid points about existing Quantitative Microbial Risk Assessments (QMRA) and their basis on sometimes invalid assumptions. The example from the literature of Listeria growth rates in raw milk being significantly lower than pasteurized milk for instance clearly shows the assumption of similar growth rates for Listeria in raw milk and pasteurized milk to be questionable if not invalid. The pilot study reported in this manuscript addresses the issues associated with previous assumptions and provides compelling support for this argument. I believe this paper should be published because there is a definite need for updating and reassessing current QMRAs as more scientific information becomes available as the authors suggest.

Suggestion
Although this work is concerned with raw milk the authors might want to mention that there are other QMRAs associated with other foods which may need to subjected to this level of scrutiny and there also needs to be some sort of formal mechanism for QMRA reevaluation. A system is in place for the Pasteurized Milk Ordinance (PMO) which changes can be made by proposal submissions at the biennial National Conference of Interstate Milk Shippers.

***Response:*** *Thank you for your comments. We will consider your helpful suggestion to propose changes at the biennial National Conference of Interstate Milk Shippers.*

*We also added this statement to the Discussion section (lines 358-361 in the manuscript revision, with all changes accepted): ‘SRA leaders seek to encourage others to apply the SRA RAQT in both review* *of completed QMRAs for other foods and water, as well as in planning for future risk analysis projects, with the goal of developing a culture of full disclosure and quality analysis.’*
